# Tunable Fermi Level Alignment in TMD Contacts via Semimetallic Bi–Sb Alloys

**DOI:** 10.1021/acsomega.6c03197

**Published:** 2026-07-30

**Authors:** Chi-Chun Cheng, Henry J. H. Chen, Chang-Hong Shen, Yu-Lun Chueh, Ying-Hao Eddie Chu, Yen-Fu Lin, Sun-Zen Chen

**Affiliations:** † Department of Electrical Engineering, National Tsing Hua University, Hsinchu 30013, Taiwan; ‡ Department of Electrical Engineering, National Chi Nan University, Puli Township, Nantou 545301, Taiwan; § College of Semiconductor Research, National Tsing Hua University, Hsinchu 30013, Taiwan; ∥ Department of Materials Science and Engineering, National Tsing Hua University, Hsinchu 30013, Taiwan; ⊥ Department of Physics, National Chung Hsing University, Taichung 40227, Taiwan; # Center for Nanotechnology, Materials Science and Microsystem, National Tsing Hua University, Hsinchu 30013, Taiwan

## Abstract

Atomically thin two-dimensional semiconductors, such as transition metal dichalcogenides (TMDs), are ushering in a new era beyond silicon-based technology. However, the persistent Fermi level pinning (FLP) effect, caused by metal-induced gap states (MIGS), creates an undesired Schottky barrier at the metal–semiconductor interface, leading to high contact resistance and degraded device performance. To broaden the applicability of TMD materials, it is crucial to establish a simple method to tune and align the Fermi levels (*E*_F_) of contact metals with those of TMDs, particularly as TMD-based devices continue to develop. Herein, we propose a strategy that uses semimetal alloys, such as bismuth–antimony (Bi–Sb), to modify the *E*_F_ of contact metals. These alloys not only eliminate MIGS-related contact issues but also enable tunable *E*_F_ tailored to different TMD materials. Our results reveal that the performance of MoS_2_ and WS_2_ field-effect transistors strongly correlates with the *E*_F_ of their contact alloys, which vary with the Bi–Sb alloy composition. The optimized device configuration exhibits a Schottky-barrier-free interface with minimal contact resistance, resulting from *E*_F_ alignment between the contact metal and the semiconductor. For MoS_2_ devices, Bi_0.03_Sb_0.97_ achieves a contact resistance of 530 Ω·μm, and a mobility of 50 cm^2^/V·s. Beyond mitigating the effects of MIGS and FLP, this work introduces a novel approach to energy level alignment, thereby broadening the scope of applications for 2D materials.

## Graphical abstract

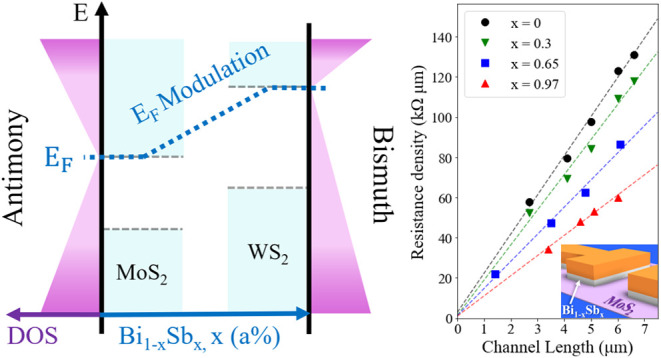

## Introduction

Two-dimensional (2D) semiconductors have attracted tremendous interest over the past decade as promising candidates for next-generation nanoelectronic technologies.^1,2^ Among these, transition metal dichalcogenides (TMDs)^3–5^ are particularly appealing due to their unique properties compared to traditional 3D counterparts. Their atomically thin geometry enables excellent electrostatic control, allowing high current densities with reduced power consumption.^6–8^ These advantages provide strong motivation to employ TMDs in “More-than-Moore” device architectures and in beyond-silicon electronics.^9,10^

Despite their promise, TMD-based devices still encounter several fundamental challenges^11,12^ before achieving widespread adoption. One of the most critical issues is the Fermi level pinning (FLP) effect at metal–semiconductor (MS) contact interfaces in van der Waals–stacked TMD structures, primarily induced by metal-induced gap states (MIGS).^21^ This FLP effect results in an uncontrollable Schottky barrier height^13–15^ and elevated contact resistance,^16^ creating a major bottleneck for high-performance TMD electronics. Addressing the FLP effect and MIGS is therefore essential for realizing reliable MS contacts.

To mitigate the FLP effect, numerous strategies have been explored, such as inserting buffer layers to suppress wave function hybridization at the interface. Approaches including van der Waals interlayers,^17,18^ ultrathin insulating layers,^19^ interface engineering, and localized doping^20^ have shown partial success; however, these methods often introduce fabrication complexity or lack compatibility across different TMD systems. A universal and straightforward method for aligning the contact electrode’s Fermi level (*E*_F_) with various TMD semiconductors remains highly desirable.

Semimetals provide a promising direction due to their intrinsically low density of states (DOS) at *E*_F_, which suppresses MIGS and can reduce or even eliminate the Schottky barrier height.^21–24^ The key is to align the *E*_F_ of the semimetal with the TMD conduction band maximum (CBM), thereby minimizing MIGS. Although previous studies have shown that both bismuth and antimony form atomically sharp, chemically clean, and MIGS-suppressed interfaces with TMDs, enabling ultralow contact resistance and stable carrier injection,^22–24^ their fixed *E*_F_ values and the limited availability of suitable semimetals restrict the applicability of this approach. This motivates the search for contact materials whose *E*_F_ can be systematically tuned, enabling energy-level alignment (ELA) tailored for a broad range of TMD semiconductors.

In this context, bismuth–antimony (Bi–Sb) alloys emerge as a particularly promising class of materials. Beyond their conventional classification as group-V semimetals, Bi, Sb, and their alloys Bi_1–_ *_x_* Sb*_x_* are well-established members of the topological materials family, exhibiting band inversion, Dirac-like dispersion, and composition-dependent electronic structures. Seminal theoretical and experimental studies—including topological quantum chemistry analyses and first-principles calculations^25–30^ —have shown that Bi–Sb alloys retain a semimetallic DOS profile (Figure S1) while exhibiting a continuously tunable *E*_F_ spanning the values of pure Bi and pure Sb. Such tunability arises from the hybridization and evolution of the *L*-point band extrema, in which small overlaps or inverted gaps allow *E*_F_ to shift with alloy composition without driving the system into a high-DOS metallic phase.^31,32^

From a device-engineering perspective, these characteristics are particularly advantageous. The low DOS at *E*_F_ suppresses MIGS and mitigates the FLP effect. At the same time, the continuous shift of *E*_F_ with alloy composition enables the contact work function to be tailored for optimal band alignment with different TMD CBM, as schematically illustrated in Figure 1a. Taken together, Bi–Sb alloys offer both *E*_F_ tunability and MIGS suppression—two properties rarely achieved simultaneously in traditional metal electrodes. These features suggest that Bi–Sb alloys could serve as a universal and composition-tunable semimetal contact strategy, enabling effectively barrierless or ohmic-like interfaces across diverse TMD materials.

**Figure 1:**
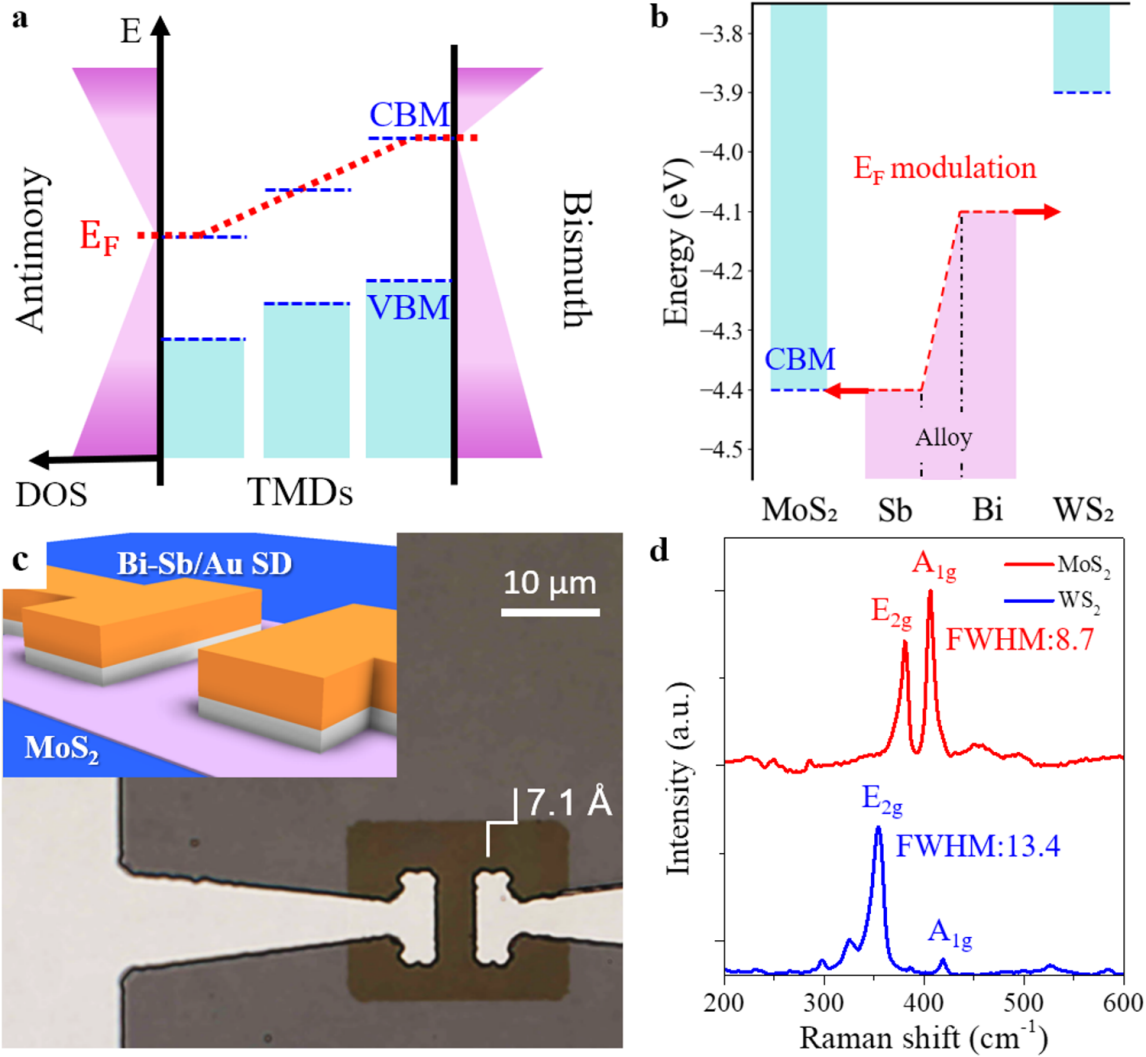
(a) Schematic diagram of utilizing semimetallic Bi–Sb alloys for energy band alignments. The *E*_F_ of the alloys can shift between the *E*_F_ of bismuth and antimony for certain TMDs. (VBM denotes the valence band maximum, and CBM denotes the conduction band minimum). (b) The energy band of Bi, Sb, MoS_2_, and WS_2_.^21^ According to theoretical predictions, an alloyed MS contact could provide better *E*_F_ matching between the contact metal and the CBM of the TMD.^21^ MoS_2_ favors the contacts with a larger amount of antimony, whereas WS_2_ prefers alloys richer in bismuth. (c) Optical image of TMDs-FET in this study after defining the active area. The inset figure shows the schematic device structure; the electrodes, i.e., marked as SD, are composed of 20 nm Bi–Sb alloy contact with 40 nm Au metal capping. (d) Raman spectra of MoS_2_ and WS_2_.

To verify this hypothesis, we fabricated a series of MoS_2_ and WS_2_ back-gated field-effect transistors (FETs) using Bi_1–_ *_x_* Sb*_x_* alloys with varying compositions as contact metals, to determine whether *E*_F_ modulation via alloy composition, as shown in Figure 1b, can improve band alignment and enhance device characteristics. Hence, in this study, we demonstrate that *E*_F_ engineering via Bi–Sb alloying provides a practical and versatile route toward optimizing MS contacts in TMD-based electronics, with an emphasis on the electronic consequences of Fermi-level modulation through alloy composition, rather than on structural imaging of the interface, which has already been extensively characterized for pure Bi and Sb contacts in prior studies. Given its versatility and simplicity, this methodology holds significant potential to enhance the electrical performance of various 2D semiconductors, paving the way for their widespread integration into future electronic applications.

## Results and Discussion

To evaluate the practical impact of Bi–Sb contact alloys on device performance, we fabricated back-gated field-effect transistors (FETs) utilizing LPCVD-grown MoS_2_ and WS_2_ as channel materials. The device architecture, depicted in Figure 1c, consists of a Si/SiO_2_ (50 nm) substrate with 20 nm-thick Bi–Sb alloy contacts capped by 40 nm Au electrodes to prevent oxidation. A standard Cr (0.5 nm)/Au (40 nm) contacted device was fabricated as a control sample. Raman spectroscopy confirms the structural integrity and high quality of the as-grown TMD films (Figure 1d), ensuring that subsequent electrical differences originate from the contact interface rather than channel degradation.

The core concept of this study relies on the continuous modulation of the Fermi level (*E*_F_) via alloying. To quantify this effect, we investigated the electronic properties of Bi_1–*x*_Sb_*x*_ alloys with varying compositions (*x* = 0.30, 0.65, and 0.97). Elemental compositions were verified by energy-dispersive X-ray spectroscopy (EDS) and X-ray photoelectron spectroscopy (XPS). As shown in Figure 2a, the XPS spectra exhibit a clear evolution in peak intensity; the Bi_0.70_Sb_0.30_ sample shows a pronounced Bi signal, whereas the Bi_0.03_Sb_0.97_ sample is dominated by Sb characteristics.

**Figure 2:**
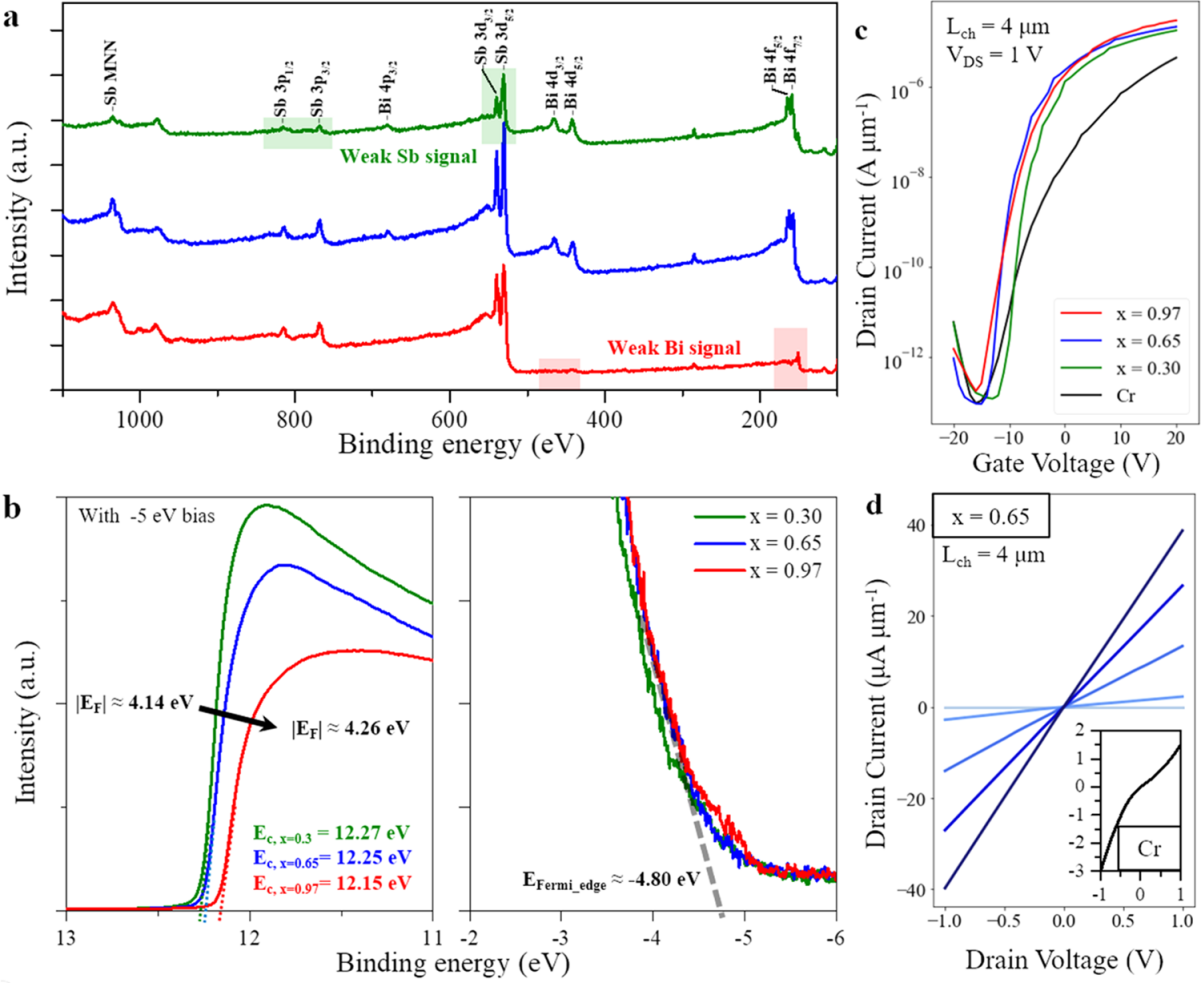
Electronic property characterizations of Bi_1–*x*_Sb*_x_* alloys, Bi_0.70_Sb_0.30_, Bi_0.35_Sb_0.65_, and Bi_0.03_Sb_0.97_. (a) X-ray photoelectron spectroscopy (XPS) spectra of the three studied alloys. Prominent peaks corresponding to the Bi and Sb orbitals are labeled. The alloy with *x* = 0.30 (green curve), Bi_0.70_Sb_0.30_, shows relatively weaker signals from Sb orbitals than the others, while the alloy with *x* = 0.97 (red curve), Bi_0.03_Sb_0.97_, exhibits negligible Bi signals. (b) Ultraviolet photoelectron spectroscopy (UPS) measurements using a He I (21.21 eV) source under a bias voltage of −5 eV. The left one magnifies the second electron cutoff energy (*E*_c_) regions, and the right one enlarges the *E*_Fermi_edge_ regions. Also listed are their calculated |*E*_F_| values, derived from the equation in the main text, which range from 4.14 to 4.26 eV. (c) Transfer characteristic curves of the studied MoS_2_ FETs under a *V*_DS_ of 1 V. (d) Output characteristics of MoS_2_ FETs with Bi_0.35_Sb_0.65_ contacts (*V*_G_: −15 to 30 V in 5 V steps), exhibiting a typical linear ohmic-like behavior. The inset shows the Cr-employing counterpart at *V*_G_ = 30 V, a nonlinear behavior in contrast.

Crucially, the modulation of the alloy work function (WF) was directly observed using ultraviolet photoelectron spectroscopy (UPS). The work function was extracted from the secondary electron cutoff energy (*E*_c_) using the relation WF = *E*_He_ – (*E*_c_ – *E*_Fermi_edge_), where *E*_He_ is the He I excitation energy (21.21 eV). For metals and semimetals, the WF is approximately given by the energy difference between the vacuum level and the Fermi level (WF ≈ *E*_vacuum_ – *E*_F_). Hereafter, the Fermi-level position (|*E*_F_|) discussed in this work refers to the effective work function extracted from UPS measurements. As presented in Figure 2b, the cutoff energy shifts systematically with increasing Sb composition, while the Fermi edge (*E*_Fermi_edge_) remains constant at approximately −4.80 eV. Consequently, the extracted |*E*_F_| range from 4.14 to 4.26 eV as the amount of Sb increases from 0.3 to 0.97, showing a composition-dependent behavior. This trend is consistent with theoretical predictions and confirms that alloying Bi with Sb enables fine-tuning of the |*E*_F_| position while maintaining semimetallic character. This continuous tunability is the prerequisite for achieving barrier-free contact formation across different TMD semiconductors.

Having established the tunable nature of the Bi–Sb alloys, we evaluated their performance as electrical contacts for MoS_2_ FETs. Figure 2c,d present a direct comparison between MoS_2_ FETs employing Bi–Sb alloy contacts and control devices fabricated with a conventional 0.5 nm Cr adhesion layer capped by 40 nm Au. As shown in the transfer characteristics (Figure 2c), devices with alloy contacts exhibit substantial improvements in both on-current and field-effect mobility compared to the Cr/Au control. Specifically, the output characteristics in Figure 2d further highlight this distinction: while the Bi–Sb alloyed devices show perfectly linear, ohmic-like behavior, the Cr-contacted control devices exhibit a highly nonlinear output-curve relationship in the inset. This nonlinearity, coupled with a significantly lower on-current, is indicative of a substantial Schottky barrier and strong Fermi level pinning typically observed in conventional metal/TMD interfaces. In contrast, the linear response over the gate voltage range from −15 to 30 V of the Bi_0.35_Sb_0.65_ alloy contact device, as an example, demonstrates that the contact barrier is effectively mitigated through precise energy level alignment and the suppression of MIGS, enabling superior carrier injection efficiency.

For a more in-depth study of the relationship between the MS interfaces and alloy compositions, we extracted the contact resistance (*R*_C_) of devices with alloy contact by using the transfer length method (TLM, a demonstration device shown in Figure 1c). The total resistance is given by *R*_total_ = *R*_channel_ + 2*R*_C_, where *R*_total_ is the total resistance, *R*_channel_ represents the channel resistance, which is a function of length. 2*R*_C_ corresponds to twice the contact resistance, which is the *y*-intercept of the linear fit to the data. As depicted in Figure 3a, for a 2D carrier density (*n*_2D_) of 1.42 × 10^13^ cm^–2^, the *R*_C_ decreases as the Sb content increases. The device with a Bi_0.03_Sb_0.97_ contact exhibits an *R*_C_ of 530 Ω·μm, the lowest among the studied MS interfaces. These results demonstrate that controlling the compositions of Bi–Sb alloys can tune the characteristics of the contact interfaces of MS junctions.

**Figure 3:**
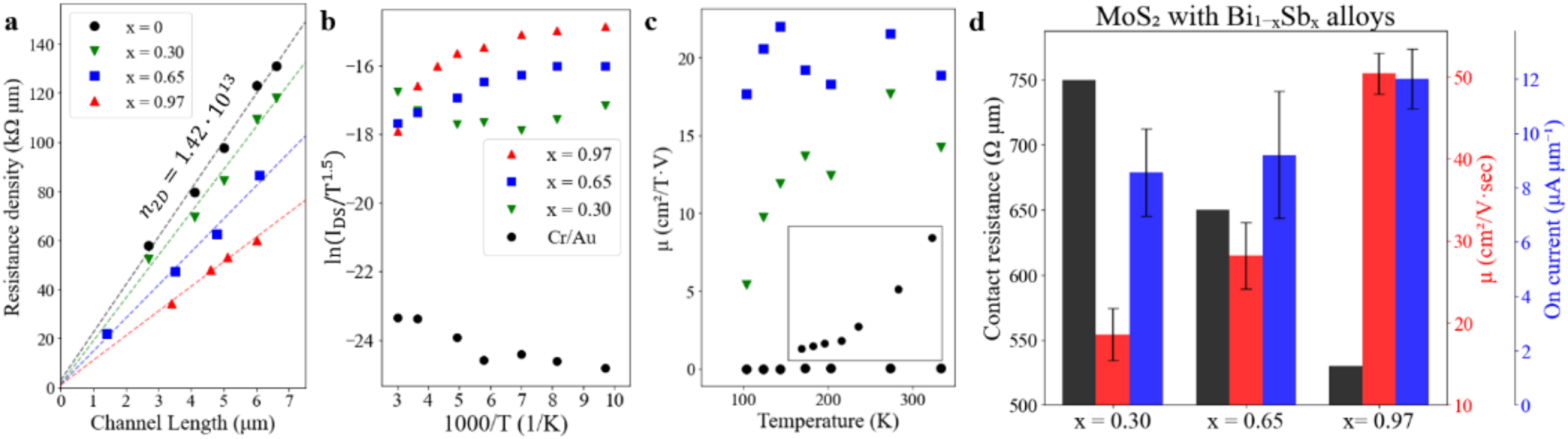
Electronic characterization of MoS_2_devices composed of Bi_1–*x*_Sb*_x_* and Cr contacts. (a) Transfer length method (TLM) characteristics of the devices with Bi_1–*x*_Sb_*x*_ contact devices. Note that *x* = 0 corresponds to pure bismuth contacts, and the y-intercepts indicate twice the contact resistance. (b) Arrhenius plots of MoS_2_ FETs that are composed of ohmic-like Bi_1–*x*_Sb_*x*_ contacts and Schottky Cr contact counterparts. (c) Temperature-dependent carrier mobility of FETs with two different Bi–Sb-alloyed contacts and that of a Cr-contacted control device. Devices employing Bi_1–*x*_Sb_*x*_ alloys maintain stable mobility over a wide temperature range, indicating the absence of Schottky-type barriers that would otherwise make carrier injection temperature-dependent. (d) Device reproducibility of the MoS_2_ FETs with Bi–Sb alloy contacts. Each data point represents measurements from 30 devices. The Bi_1–*x*_Sb_*x*_ alloy-contacted devices exhibit improved device characteristics with the increase of Sb, as expected.

To further evaluate barrier properties of the MS interfaces, temperature-dependent current measurements were conducted to extract the interface barrier height. The current behavior can be described using the Schottky diode equation

$$J_{\mathrm{DS}}=A^{*}\cdot T^{1.5}\cdot \exp\left(-\frac{{q\Phi }_{\mathrm{SB}}}{k_{B}T}\right)\cdot \left[\exp\left(\frac{qV_{\mathrm{DS}}}{k_{B}T}\right)-1\right]$$

where A* is the Richardson constant, *T* is the temperature, and Φ_SB_ represents the Schottky barrier height (SBH). The SBH was extracted from Arrhenius plots using the relationship

$$\ln\left(I_{\mathrm{DS}}/T^{1.5}\right)\propto -q\Phi_{\mathrm{SB}}/k_{B}T$$

As shown in Figure 3b, the device with Cr contact exhibits a typical negative slope in the Arrhenius plot, presenting a Schottky-dominated MS interface. In sharp contrast, the FETs with Bi–Sb alloy contacts exhibit positive slopes or nearly flat characteristics as temperature decreases. This behavior signals the effective elimination of the Schottky barrier, indicating that the transport is dominated by tunneling or is effectively Schottky-barrier-free due to the suppression of MIGS and precise ELA. Notably, the suppression of the FLP effect is a function of the composition of the Bi–Sb alloys, and the Bi_0.03_Sb_0.97_ alloy exhibits the most pronounced trend in suppressing the FLP effect with the most positive slope in the Arrhenius plot. It should be noted that, for contacts approaching the ohmic limit, the extracted slope does not represent a true thermionic Schottky barrier height but instead reflects suppressed thermally activated transport, consistent with tunneling-dominated injection.

This conclusion is further supported by the temperature-dependent mobility analysis shown in Figure 3c, which shows that the mobility values of devices with Bi–Sb contacts are much higher than those of the Cr control device. The Cr-contacted device exhibits a drop in carrier mobility with decreasing temperature, as shown in the inset. This is typical of a Schottky interface, a hallmark of barrier-limited injection where carriers lack sufficient thermal energy to overcome the interface barrier. In contrast, FETs with alloy contacts maintain high and relatively stable mobility across a wide temperature range, i.e., from room temperature to 100 K, indicating the effective elimination of the Schottky barrier.

Finally, to demonstrate reproducibility, we statistically analyzed key performance metrics from 30 devices (Figure 3d). The results confirm that device characteristics are strongly dependent on the alloy composition. Among the three alloys studied, devices employing Bi_0.03_Sb_0.97_ as the contact metal exhibit the lowest contact resistance of 530 Ω·μm. In addition, these devices consistently show superior performance in other critical metrics, such as mobility and on-current. The observed performance variation of MoS_2_ FETs with different Bi–Sb alloy contacts can be rationalized by the tunability of the alloys’ |*E*_F_|.

To demonstrate that the Fermi level modulation in ELA capability of Bi–Sb alloys is not limited to a single material system but is a universally applicable strategy for TMDs, we extended our investigation to WS_2_. Figure 4a and b show the calculated density of states for pure bismuth and antimony, yielding |*E*_F_| of approximately 4.1 and 4.4 eV, respectively.^21^ The UPS measurements in Figure 4b reveal how *E*_F_ is systematically modified by the Bi–Sb composition. WS_2_ typically has a higher conduction band minimum (CBM) than MoS_2_, which theoretically necessitates a contact material with a smaller work function (i.e., a shallower |*E*_F_|) to achieve optimal alignment.

**Figure 4:**
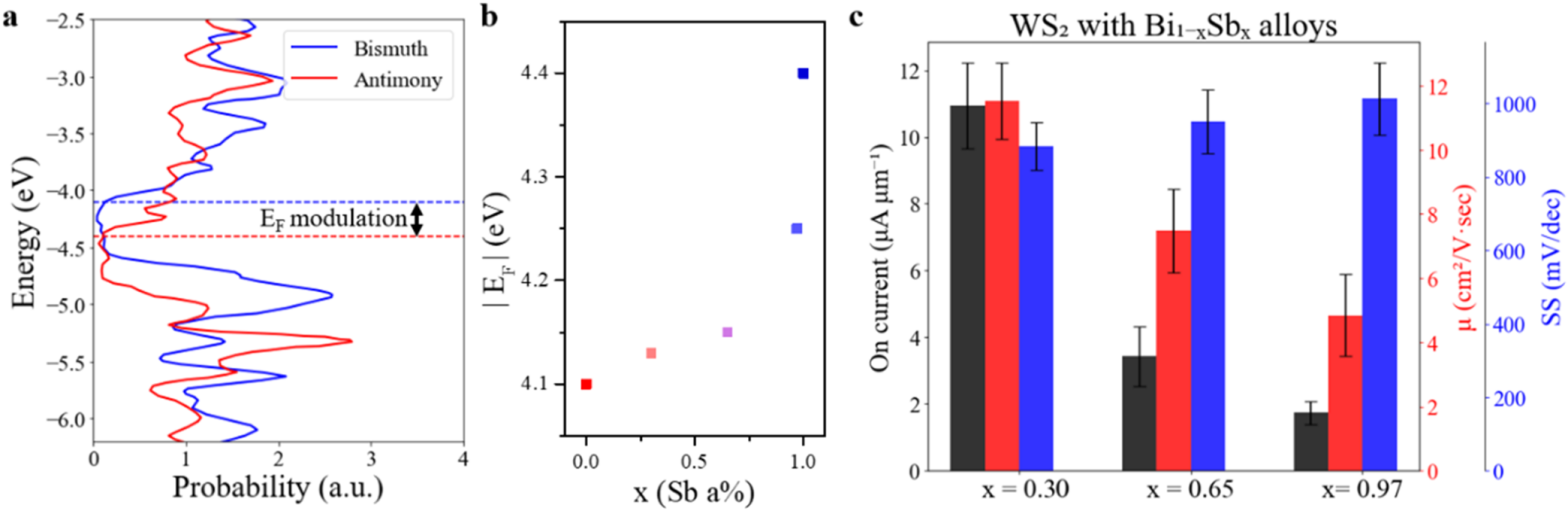
Band structures of examined semimetals and Device characteristics of WS_2_FETs with Bi_1–*x*_Sb*_x_* contact. (a) Density of states for pure bismuth and antimony. The dashed lines indicate their respective Fermi levels.^26–30^ (b) The UPS results show that |*E*_F_| increases monotonically with increasing Sb. Confirming that the |*E*_F_| of Bi–Sb alloy shifts with Bi and Sb and can be modified by the content. (c) Device performance summary of WS_2_ FETs employing Bi–Sb alloy contacts. Each data point represents the measurements of 30 devices. As shown, device performance improves as the Bi content increases.

Figure 4c illustrates the application of Bi–Sb alloy contacts to WS_2_ FETs. In stark contrast to the MoS_2_ results, where Sb-rich alloys yielded the best performance, the electrical characteristics of WS_2_ devices improve as the bismuth content increases. As summarized in Figure 4c, devices with Birich contacts (Bi_0.70_Sb_0.30_) consistently exhibit superior performance metrics, including higher on-currents, higher field-effect mobility, and reduced subthreshold swings, compared to their Sb-rich counterparts.

This “trend reversal” between MoS_2_ and WS_2_ provides the most compelling experimental validation of our band engineering hypothesis. As analyzed in Figure 4a,b, pure Bismuth has a lower work function (∼4.14 eV) than pure Antimony (∼4.26 eV). Consequently, Birich alloys exhibit a higher-lying Fermi level that more closely aligns with the shallower CBM of WS_2_. Conversely, the deeper CBM of MoS_2_ benefits from the lower-lying Fermi level provided by Sb-rich alloys. These improvements are consistent with the *E*_F_ modulation results in Figure 1b. By simply adjusting the alloy stoichiometry, we can switch from an optimal contact for MoS_2_ to an optimal contact for WS_2_ without changing the metallization material system or fabrication process. This demonstrates that the alloying strategy effectively decouples the choice of contact metal from the intrinsic constraints of FLP effect, offering a robust pathway to maximize the performance of diverse 2D semiconductor devices.

## Conclusions

In summary, we have successfully demonstrated a versatile strategy for modifying the Fermi level (*E*_F_) of metal–semiconductor contacts via alloying of semimetals. By employing Bi–Sb alloys as a model system, we confirmed that the contact *E*_F_ can be continuously tuned between the intrinsic levels of bismuth and antimony. This continuous tunability is critical for achieving precise energy level alignment with the conduction bands of various TMD semiconductors.

Our experimental results on MoS_2_ and WS_2_ FETs validate this concept, showing that device performance—including carrier mobility and contact resistance—is strictly dependent on the alloy composition. Specifically, the significant reduction in Schottky barrier height and the realization of Ohmic-like characteristics in both material systems provide strong evidence that the Fermi level pinning effect can be effectively mitigated by this alloying approach.

More importantly, this work establishes a proof of concept for semimetallic alloy contacts. Rather than merely optimizing a specific contact for a single material, we present a generalized methodology that can be extended to other semimetallic systems and 2D materials. This approach provides a simple yet powerful degree of freedom for interface engineering, enabling the development of high-performance, energy-efficient 2D electronics with tailored contact properties.

## Methods

### Ultraviolet Photoelectron Spectroscopy (UPS) and X-ray Photoelectron Spectroscopy (XPS)

Ultraviolet photoelectron spectroscopy (UPS) and X-ray photoelectron spectroscopy (XPS) measurements were performed to determine the work function and chemical composition of Bi–Sb alloy films. To avoid charging effects during photoelectron measurements, highly doped Si substrates were used as conductive substrates. Bi_1–_ *_x_* Sb_*x*_ alloy films with a thickness of approximately 100 nm were deposited on the doped Si substrates under identical conditions to those used for device fabrication.

XPS measurements were conducted to verify the elemental composition and chemical states of the Bi–Sb alloy films. Core-level spectra of Bi and Sb were collected to confirm the alloy composition and uniformity. UPS measurements were carried out using a He I ultraviolet light source (photon energy of 21.21 eV) to probe the valence band region and determine the sample work function. A negative sample bias was applied during UPS measurements to clearly resolve the secondary electron cutoff. The work function was extracted from the energy difference between the secondary electron cutoff and the Fermi edge.

All UPS and XPS measurements were performed under ultrahigh vacuum conditions using a combined ESCA system, ensuring minimal surface contamination during analysis. The measured work function values were used to correlate the alloy composition with Fermi-level tuning and band alignment in TMD-based devices.

### LPCVD of Monolayer MoS_2_

MoS_2_ monolayer crystals were grown using low-pressure chemical vapor deposition on sapphire substrates. The substrates were preannealed at 1000 °C in 2000 standard cubic centimeters per minute (sccm) of oxygen (∼10 Torr) for 2 h and then cooled to room temperature, which took approximately 4 h. After cleaning with acetone, isopropanol, and DI water, the substrates were placed face-up at the downstream in the furnace. The quartz boat carrying 3 mg precursor molybdenum trioxide (MoO_3_, 99.98%) powder was set at the center of the furnace, and another precursor, 10 mg sulfur powder, was put at the upstream of the furnace. Before growing, the whole furnace was first purged with 500 sccm argon at 100 °C for 3 min. Then, the argon was set to a flow rate of 60 sccm as the carrier gas, and the chamber pressure was maintained at 35 Torr. The temperatures of MoO_3_ and sulfur were raised to 650 and 160 °C, respectively, for a 10 min MoS_2_ growth period.

### LPCVD of Monolayer WS_2_

WS_2_ monolayers were grown using a method similar to that for MoS_2_. The annealed sapphire substrate was placed 0.5 cm away from the tungsten trioxide (WO_3_) powder precursor, i.e., 0.5 g, on the quartz boat. The boat was set in the center of the furnace, and sulfur powders of 10 mg were at the upstream. After purging, the temperatures of WO_3_ and sulfur were raised to 850 and 160 °C, respectively. The growth environment was controlled at 35 Torr with argon at 200 sccm and hydrogen at 20 sccm for 10 min.

### Transfer of Monolayer TMDs onto Si Substrates

Before fabricating the back-gate TMD devices, the grown TMD materials were wet transferred onto p++-silicon substrates with a 50 nm silicon oxide layer. The wet transfer process utilized a 3% polycarbonate (PC) chloroform solution as the transferring polymer film. First, the PC solution was spin-coated onto the TMD materials-grown Si substrate at 3000 rpm, followed by dipping the sample into DI water to peel off the PC/TMDs film. To reduce the water residue, the PC/TMDs film was transferred onto an isopropanol-water solution using a filter paper. Then, the 50 nm silicon oxide-grown Si substrate was used to pick up the PC/TMDs film, and the substrate was dipped into pure isopropanol to remove the residual water. The sample was later baked on a hot plate at 85 °C for 40 min and soaked in chloroform at 65 °C for 12 h to remove the PC polymer film. This was followed by sequential dipping into acetone and isopropanol solutions at 65 °C to dissolve the chloroform. Finally, the TMDs/SiO_2_/Si sample was baked in a low-vacuum furnace at 180 °C with an argon flow rate of 100 sccm for 1 h.

### Device Fabrication and Characterization

The source/drain electrodes were patterned via a digital lithography process with a bilayer resist structure, i.e., LOR/S1813. Then, a contact layer (20 nm for Bi–Sb alloys or 0.5 nm for Cr) was deposited by a thermal evaporator at a rate of 0.2 Å/s under 10–6 Torr, followed by a 40 nm Au capping layer deposited at a rate of 1 Å/s. After lift-off and soaking in a 65 °C PG remover for 20 min, the samples were annealed in a low-pressure (∼10^–2^ Torr) furnace at 180 °C under 100 sccm argon for 2 h. The characterization of electrical properties was conducted in a low-vacuum (∼10^–2^ Torr) probe station using a Keysight 4200. Raman spectroscopy (Thermo DXR, 523 nm laser, 10 mW) was used to investigate the crystal quality of MoS_2_ and WS_2._

## Supplementary Material



## Notes

Additional experimental details are provided, including the density of states (DOS) of Bi–Sb alloys, details of LPCVD growth and AFM characterization of monolayer MoS_2_ and WS_2_, Schottky barrier extraction, and SEM/EDS characterization of Bi–Sb alloy films (PDF)
