# Supplementary material for: Tunable Fermi Level Alignment in TMD Contacts via Semimetallic Bi–Sb Alloys
Source: ACS Omega. 2026 Jul 30;11(31):47032–8. doi: 10.1021/acsomega.6c03197 (PMC13470917; doi:10.1021/acsomega.6c03197)
Supplement: Supplementary file 1 [file ao6c03197_si_001.pdf]

Tunable Fermi Level Alignment in TMD Contacts via Semimetallic Bi–Sb Alloys

Chi-Chun Cheng[1], Henry J. H. Chen[2], Chang-Hong Shen[3], Yu-Lun Chueh[4], Ying-Hao Eddie Chu[4], Yen-Fu Lin[5] and Sun-Zen Chen[6*]

[*]E-mail: szchen@mx.nthu.edu.tw

[1]*Department of Electrical Engineering, National Tsing Hua University, Hsinchu 30013, Taiwan.*

[2]*Department of Electrical Engineering, National Chi Nan University, Puli Township, Nantou, Taiwan*

[3]*College of Semiconductor Research, National Tsing Hua University, Hsinchu 30013, Taiwan.*

[4]*Department of Materials Science and Engineering, National Tsing Hua University, Hsinchu 30013, Taiwan.*

[5]*Department of Physics, National Chung Hsing University, Taichung 40227, Taiwan.*

[6]*Center for Nanotechnology, Materials Science and Microsystem, National Tsing Hua University, Hsinchu 30013, Taiwan.*

Supplementary Details

S1. Density of states of Bi–Sb semimetallic alloys from first-principles calculations

To support the experimental observation that Bi–Sb alloys retain semimetallic characteristics while exhibiting tunable Fermi-level positions, we examined density-of-states (DOS) data for representative $Bi_{1-x}Sb_x$ alloys reproduced from the publicly available Topological Materials Database [1–5]. The database provides first-principles DFT results based on the methodology reported by Vergniory *et al* [2,3]. As shown in Fig. S1, alloying Bi with Sb does not introduce a high DOS at the Fermi level despite the composition-dependent shift of E_F. Instead, the DOS near E_F remains low for the examined alloy compositions, indicating that Bi–Sb alloys preserve their semimetallic character rather than transitioning into a conventional high-DOS metallic state. These literature-based DOS results provide theoretical support for using Bi–Sb alloys as composition-tunable semimetal contacts in this work.

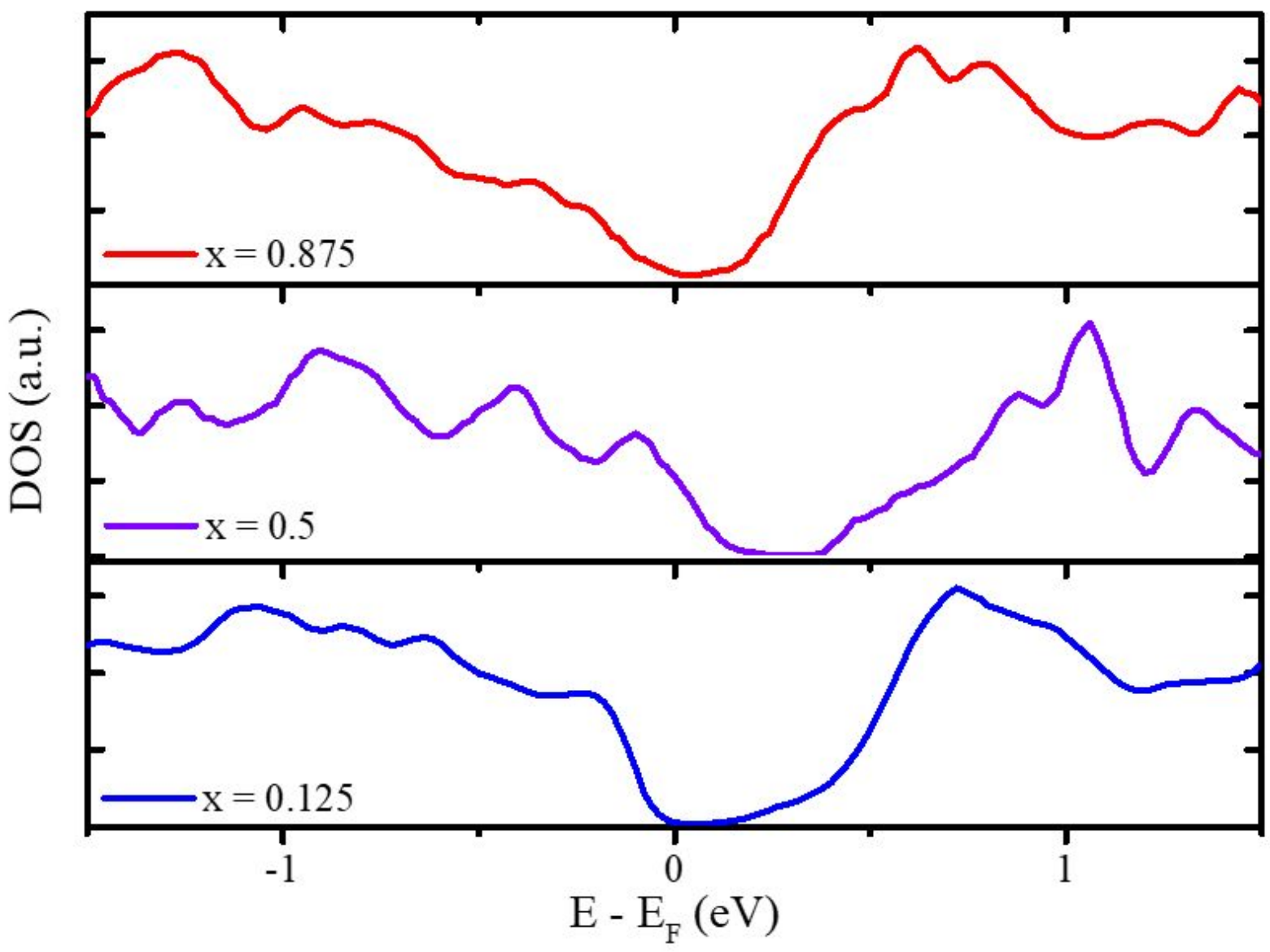

Figure S1 | Calculated density of states (DOS) of representative $Bi_{1-x}Sb_x$ alloys obtained from first-principles calculations reproduced from the literature [1–5]. The Fermi level (E_F) is set to 0 eV. Across a wide range of alloy compositions, the DOS nearby E_F remains low, indicating that Bi–Sb alloys preserve their semimetallic character despite continuous Fermi-level tuning.

S2. Low-pressure chemical vapor deposition growth of TMD crystals

Monolayer MoS_2 and WS_2 crystals used in this study were synthesized by low-pressure chemical vapor deposition (LPCVD) on sapphire substrates. Prior to growth, sapphire substrates were annealed at 1000 °C under an oxygen atmosphere to improve surface cleanliness and promote uniform nucleation. Molybdenum trioxide (MoO_3)/tungsten trioxide (WO_3) and sulfur powders were used as the metal and chalcogen precursors, respectively. Argon was employed as the carrier gas during growth. The experimental configuration is illustrated in Fig. S2, and the LPCVD furnace consists of three independently controlled heating zones. An additional inner quartz tube with a partially open downstream end was introduced to locally enhance the precursor concentration above the sapphire substrate. The growth temperature and precursor positioning were optimized to yield triangular TMD domains with lateral sizes suitable for device fabrication. The temperature settings for the three heating zones were approximately 180/530/850 °C for MoS_2 growth and 180/850/900 °C for WS_2 growth, respectively.

Optical microscopy and atomic force microscopy (AFM) were further used to characterize the morphology and thickness of the as-grown TMDs. As shown in Fig. S2, both MoS_2 and WS_2 exhibit triangular domains, which are characteristic of CVD-grown monolayer TMD crystals. AFM height profiles show step heights of approximately 7.1 Å for MoS_2 and 7.3 Å for WS_2, consistent with monolayer MoS_2 and WS_2 reported in the literature. These results confirm that the LPCVD-grown TMD crystals used in this study are monolayer domains with lateral dimensions suitable for device fabrication.

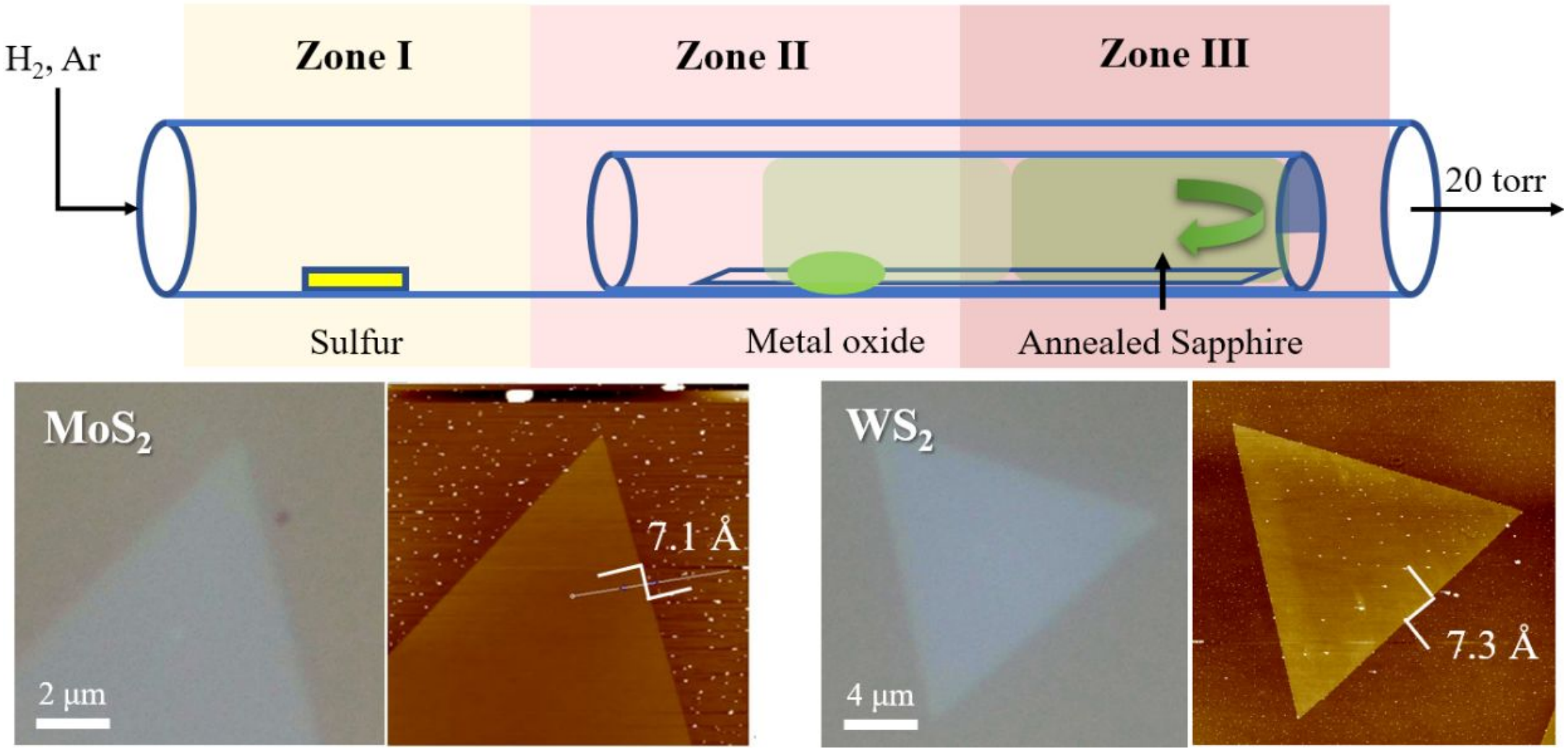

Figure S2 | Growth configuration and morphology characterization of LPCVD-grown monolayer TMD crystals. Schematic illustration of the three-zone LPCVD system used for MoS_2 and WS_2

growth, showing the positions of the sulfur precursor, metal oxide precursor, and annealed sapphire substrate. Optical microscopy images show triangular MoS_2 and WS_2 domains grown on sapphire substrates. AFM images and height profiles confirm monolayer thicknesses, with step heights of approximately 7.1 Å for MoS_2 and 7.3 Å for WS_2, respectively.

S3. Extraction of effective Schottky barrier height

The effective Schottky barrier height ($q\Phi_{SB}$) was quantitatively extracted by analyzing temperature-dependent transfer characteristics based on the thermionic emission model, which is described by the equation

$$J_{DS} = A^* \cdot T^{1.5} \cdot \exp\left(-\frac{q\Phi_{SB}}{k_B T}\right) \cdot \left[\exp\left(\frac{qV_{DS}}{k_B T}\right) - 1\right]$$

In this expression, A^* represents the effective Richardson constant, k_B is the Boltzmann constant, and T denotes the absolute temperature. By constructing Arrhenius plots of $\ln\left(I_{DS}/T^{1.5}\right)$ versus 1000/T across a range of gate voltages (V_G), the effective energy barrier was determined from the resulting slopes. The experimental results for the Bi–Sb alloy contacts indicate that the extracted effective Φ_{SB} decreases linearly with increasing gate bias, a behavior attributed to the gate-induced modulation of the semiconductor's conduction band. At the flat-band voltage condition, the Bi–Sb alloy contacts exhibit exceptionally low or negative effective barrier heights. These results stand in sharp contrast to the typical Schottky behavior and negative slopes observed in the Arrhenius plots of the Cr-contacted control devices. Such findings demonstrate that semimetallic alloy contacts effectively suppress metal-induced gap states (MIGS) and achieve precise energy level alignment, leading to the formation of intrinsically Schottky-barrier-free interfaces.

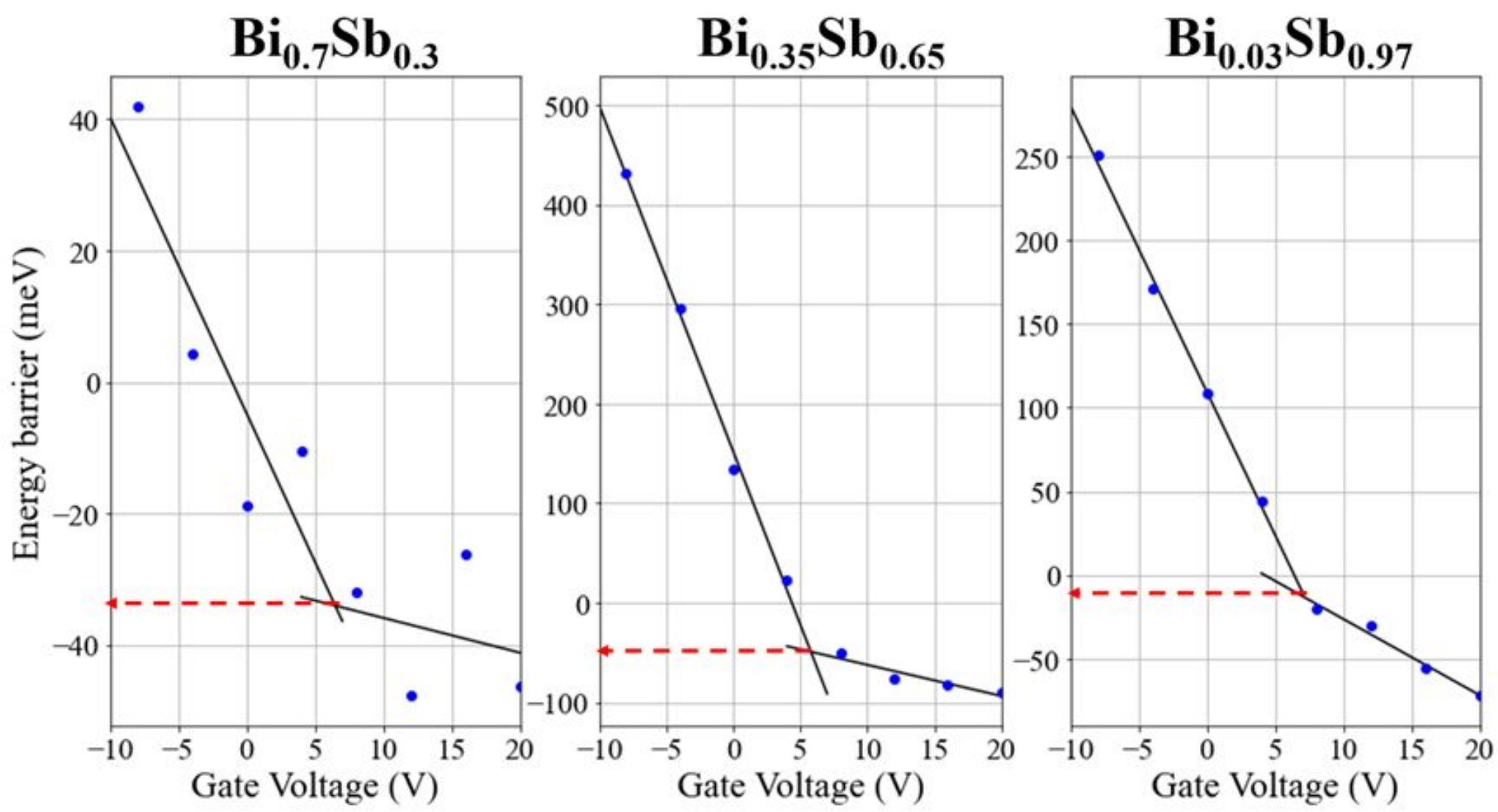

Figure S3 | Extraction of effective Schottky barrier height(Φ_{SB}). Effective Φ_{SB} as a function of gate voltage (V_G) for MoS_2 FETs employing $Bi_{1-x}Sb_x$ alloy contacts with compositions of x = 0.30, 0.65, and 0.97. The red dashed lines represent the flat-band barrier heights extracted from the slopes of temperature-dependent Arrhenius plots. The near-zero and negative values indicate the effective elimination of the Schottky barrier and the formation of Ohmic-like interfaces.

S4. SEM Characterization of the Bi–Sb Alloy Contact Layer

High-resolution top-view scanning electron microscopy (SEM) was used to characterize the surface morphology and continuity of the deposited Bi–Sb alloy contact layers (Fig. S4). At a high magnification of 100,000×, the SEM images show that the alloy films form compact and fine-grained structures. Although slight differences in surface texture are observed among different alloy compositions, both Bi-rich and Sb-rich films exhibit continuous and dense morphologies at the sub-100 nm scale. This continuous growth behavior is distinct from the discrete island-like morphology typically associated with ultrathin metal deposition through Volmer–Weber growth.

Energy-dispersive X-ray spectroscopy (EDS) was further performed to verify the elemental composition of the deposited Bi–Sb alloy films. As shown in Fig. S4c, characteristic signals from both Bi and Sb are observed for all alloy compositions, confirming the coexistence of the two constituent elements in the deposited films. In addition, the relative intensities of the Bi and Sb peaks vary systematically with the nominal alloy composition: the $Bi_{0.70}Sb_{0.30}$ sample exhibits stronger Bi-related signals, whereas the $Bi_{0.03}Sb_{0.97}$ sample is dominated by Sb-related signals. These EDS results, together with the SEM observations, support the successful formation of continuous Bi–Sb alloy contact layers with composition-dependent elemental ratios.

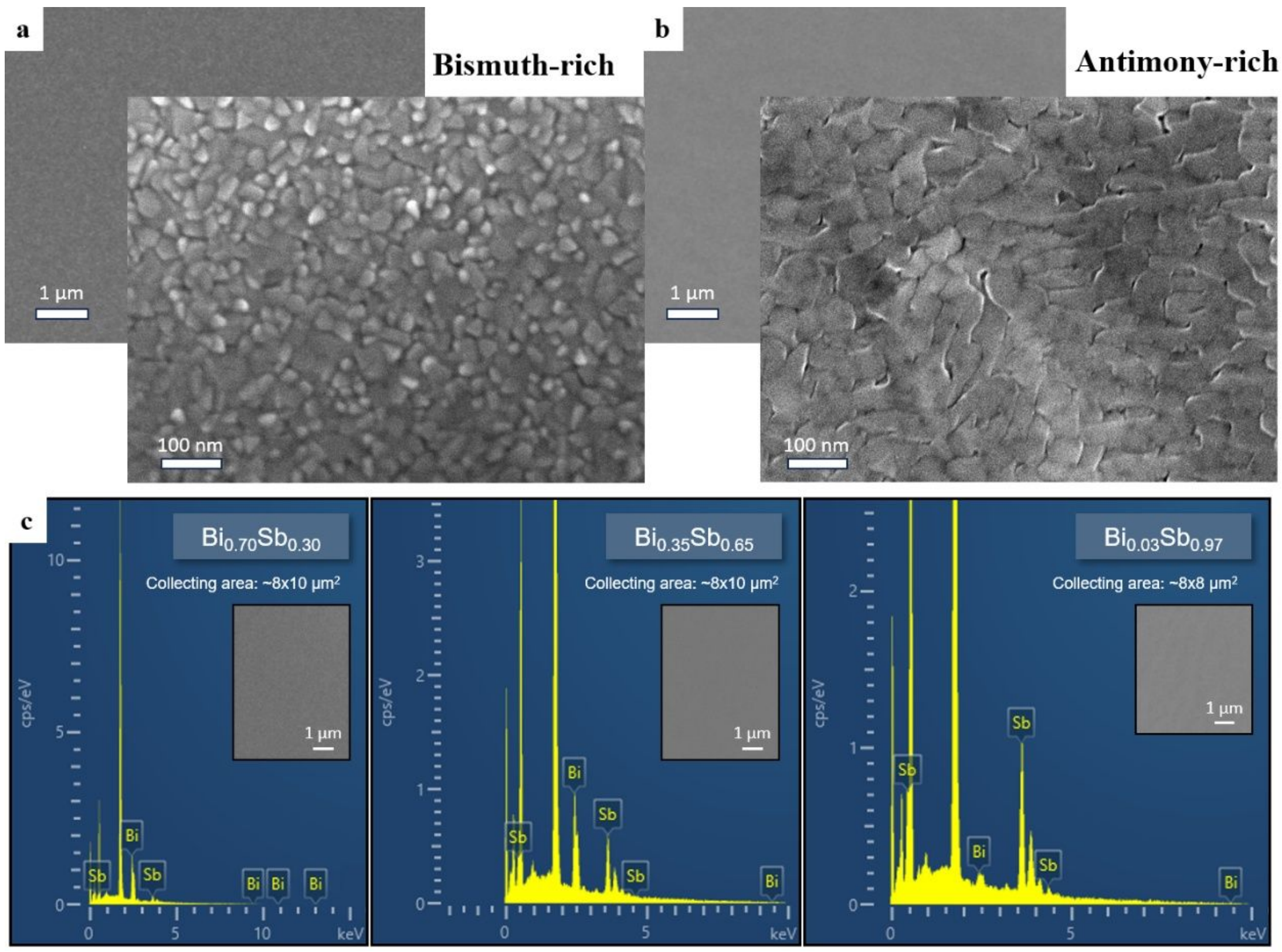

Figure S4 | SEM and EDS characterization of Bi–Sb alloy contact films. (a,b) Top-view SEM images of representative Bi-rich and Sb-rich alloy films, showing bismuth-like and antimony-like surface morphologies, respectively. The high-magnification images reveal continuous and compact film formation at the sub-100 nm scale. (c) EDS spectra collected from $Bi_{0.70}Sb_{0.30}$, $Bi_{0.35}Sb_{0.65}$, and $Bi_{0.03}Sb_{0.97}$ alloy films. The insets show the corresponding SEM images and the EDS collection areas. Characteristic Bi and Sb signals are observed, and their relative intensities vary consistently with the nominal alloy compositions, supporting the successful formation of composition-tunable Bi–Sb alloy contact layers.

References

1. Bradlyn, B., et al. "Topological Quantum Chemistry." Nature, vol. 547, 2017, pp. 298–305.
2. Vergniory, M. G., et al. "A Complete Catalogue of High-Quality Topological Materials." Nature, vol. 566, 2019, pp. 480–485.
3. Vergniory, Maia G., et al. "All Topological Bands of All Nonmagnetic Stoichiometric Materials." Science, vol. 376, 2022, p. eabg9094.
4. "Topological Material Database." TopologicalQuantumChemistry.org, http://topologicalquantumchemistry.org/.
5. "Bilbao Crystallographic Server." Cryst.EHU.es, http://www.cryst.ehu.es/.
